# Investigation of gene effects and epistatic interactions between *Akt1* and *neuregulin 1* in the regulation of behavioral phenotypes and social functions in genetic mouse models of schizophrenia

**DOI:** 10.3389/fnbeh.2014.00455

**Published:** 2015-01-29

**Authors:** Ching-Hsun Huang, Ju-Chun Pei, Da-Zhong Luo, Ching Chen, Yi-Wen Chen, Wen-Sung Lai

**Affiliations:** ^1^Department of Psychology, National Taiwan UniversityTaipei, Taiwan; ^2^Graduate Institute of Brain and Mind Sciences, National Taiwan UniversityTaipei, Taiwan; ^3^Neurobiology and Cognitive Science Center, National Taiwan UniversityTaipei, Taiwan

**Keywords:** Akt1 mutant mice, neuregulin 1 (Nrg1) mutant mice, epistatic effect, schizophrenia, behavioral phenotyping, social interaction and communication

## Abstract

Accumulating evidence from human genetic studies has suggested several functional candidate genes that might contribute to susceptibility to schizophrenia, including *AKT1* and* neuregulin 1* (*NRG1*). Recent findings also revealed that NRG1 stimulates the PI3-kinase/AKT signaling pathway, which might be involved in the functional outcomes of some schizophrenic patients. The aim of this study was to evaluate the effect of Akt1-deficiency and Nrg1-deficiency alone or in combination in the regulation of behavioral phenotypes, cognition, and social functions using genetically modified mice as a model. Male *Akt1*^+/−^, *Nrg1*^+/−^, and double mutant mice were bred and compared with their wild-type (WT) littermate controls. In Experiment 1, general physical examination revealed that all mutant mice displayed a normal profile of body weight during development and a normal brain activity with microPET scan. In Experiment 2, no significant genotypic differences were found in our basic behavioral phenotyping, including locomotion, anxiety-like behavior, and sensorimotor gating function. However, both *Nrg1*^+/−^ and double mutant mice exhibited impaired episodic-like memory. Double mutant mice also had impaired sociability. In Experiment 3, a synergistic epistasis between *Akt1* and *Nrg1* was further confirmed in double mutant mice in that they had impaired social interaction compared to the other 3 groups, especially encountering with a novel male or an ovariectomized female. Double mutant and *Nrg1*^+/−^ mice also emitted fewer female urine-induced ultrasonic vocalization calls. Collectively, our results indicate that double deficiency of *Akt1* and *Nrg1* can result in the impairment of social cognitive functions, which might be pertinent to the pathogenesis of schizophrenia-related social cognition.

## Introduction

Schizophrenia is a costly and devastating mental disorder that afflicts approximately 1 percent of the worldwide population. Clinically, schizophrenia is mainly characterized by positive symptoms, including delusions and hallucinations, negative symptoms, such as blunted emotions, anhedonia, and social isolation, and cognitive deficits, such as the impairment of executive function, attention, and working memory. Schizophrenia is a devastating mental illness with a strong genetic predisposition. Accumulating evidence emerging from human genetic studies suggests several positional candidate loci and functional candidate genes. Although the etiology of schizophrenia remains largely unclear, the influence of a genetic predisposition is of critical importance for the pathogenesis of schizophrenia. Among these candidate genes, *neuregulin 1* (*NRG1*) and *AKT1* (PKBα) appear to be promising and worth further investigation based upon human genetic studies, review articles, and genome-wide meta-analyses (Harrison and Weinberger, 2005; Schwab and Wildenauer, 2009; Kvajo et al., 2010; Emamian, 2012). *NRG1* was initially implicated in schizophrenia by a study in an Icelandic sample (Stefansson et al., 2002), and association analyses across 8p21-22 revealed highly significant associations. NRG1, a trophic factor of neuregulin family, is a ligand and a receptor for epidermal growth factor-life receptor (ErbB) 1–4 and NRG1 has a transmembrane (TM) domain that is a critical motif for forward and reverse signaling cascade (Bao et al., 2003; Falls, 2003; Mei and Xiong, 2008). Numerous roles for NRG1 in central nervous system development and function have been identified (Falls, 2003; Harrison and Law, 2006), but the precise functions of NRG1 signaling in the pathogenesis of schizophrenia remains unclear.

AKT1, a serine/threonine kinase of the AKT family, is involved in multiple biological processes. Evidence for *AKT1* as a susceptibility gene for schizophrenia was reported in Caucasian families of European descent originally and subsequently in several other populations (Emamian et al., 2004; Norton et al., 2006; Schwab and Wildenauer, 2009). Studies of the postmortem brains of schizophrenic patients (Emamian et al., 2004; Zhao et al., 2006), *Akt1*-deficient mice (Lai et al., 2006; Chen and Lai, 2011; Chen et al., 2012, 2014), and functional neuroimaging in humans (Tan et al., 2008) support the idea that genetic variations in *AKT1* or its protein have epistatic effects on the regulation of dopamine-dependent functions and antipsychotic effects. The biological functions of AKT1 and the mechanism by which it contributes to a susceptibility to schizophrenia need further investigation.

Interestingly, AKT1 is one of the downstream kinases of the NRG1 signaling pathway (Downward, 1998; Marmor et al., 2004). Recent findings indicated that NRG1 acts through ErbB2/4 in a paracrine fashion to stimulate the PI3-kinase/AKT signaling pathway (Keri et al., 2009a). An *in vitro* study also indicated that NRG1 signaling is associated with schizophrenia via the PI3K/AKT-dependent pathway (Kanakry et al., 2007). In a discordant monozygotic twins study, the twin with schizophrenia also displayed lower NRG1-stimulated AKT phosphorylation (Seres et al., 2010). A reduction of sensory gating function and decrease of NRG1-stimulated AKT phosphorylation were also reported in non-treated, first-episode schizophrenic patients (Keri et al., 2010). In non-clinical individuals, decreased habituation of arousal, NRG1-stimulated AKT phosphorylation, and anxiety were found to be related to delusional ideation (Keri et al., 2009a,b), suggesting a potential gene-gene interaction in the pathogenesis of some schizophrenic symptoms.

In addition, cognitive impairment, especially deficits in social cognition, is a core feature of schizophrenia that strongly influences the quality of life and function of people with this illness (Elvevåg and Goldberg, 2000; Green, 2006). Accumulating evidence indicated that AKT1 and NRG1 were involved in social functions of schizophrenia. For example, *NRG1* rs221533 SNP was associated with premorbid social functioning and personality development in teenagers (Walshe et al., 2012). *Nrg1* mutant mice displayed enhancement of aggressive behaviors and impairments in social recognition (O’Tuathaigh et al., 2007, 2008; Pei et al., 2014). A reduction of phospho-AKT levels in the ventral tegmental area was also reported in mice with chronic social defeat (Krishnan et al., 2008). The roles of *AKT1* and *NRG1*, either alone or their interaction, through which they might contribute to a susceptibility to social function in schizophrenia are worth further investigation.

In complement to human genetic studies, animal studies of a gene unequivocally implicated by human genetics are necessary to identify functional consequence or mechanism. Mouse models play an indispensable role in the discovery of potential drugs/treatments and provide a feasible approach to elucidate causal relationships between genes and related symptoms (Arguello and Gogos, 2006; Lai et al., 2014). Notably, it is often insufficient to use just one mouse behavioral task because each task assesses different aspects of behavioral function and fulfills different aspects of the validity criteria (Lai et al., 2014). As a first step, a set of three experiments was conducted in this study to characterize the effect of *Akt1^+/−^*, *Nrg1*^+/−^, and double deficiency on behavioral phenotypes and cognitive/social functions in mice, compared to their WT littermate controls. In Experiment 1, the general physical development, protein expression and basic brain activity were measured in these mice. In Experiment 2.1, basic behavioral phenotypes were evaluated in these mice. Cognitive and social functions were examined in Experiment 2.2. Based on the findings in Experiments 1 and 2, social interaction and social communication were further investigated in Experiment 3.

## Materials and methods

### Animals

All mice, *Akt1* heterozygous (*Akt1*^+/−^) mice, *Nrg1* heterozygous (*Nrg1*^+/−^), *Akt1-Nrg1* double heterozygous (double mutant) mice, and their wild-type (WT) littermates used in this study were produced by *Akt1*^+/−^ and *Nrg1*^+/−^ breeding pairs of both sexes in a C57BL/6 genetic background (backcrossed over 10 generations). Conventional Akt1 and Nrg1 transmembrane-domain single and double mutant mice were used and genotyped as described previously (Cho et al., 2001; Pei et al., 2014). The Akt1 mutant mice were originally created by truncation of the coding exons 4–8 and used as an experimental tool to mimic the reduction of AKT1 protein in some patients with schizophrenia (Cho et al., 2001; Emamian et al., 2004; Lai et al., 2006). The Nrg1 mutant mice were generated by truncation of transmembrane-domain of *Nrg1* gene to mimic a NRG1 deficiency in some schizophrenic patients (Stefansson et al., 2002; Pei et al., 2014). Both mutant mice were validated as a genetic mouse model of schizophrenia (Lai et al., 2006; Chen et al., 2014; Pei et al., 2014). After weaning on postnatal day (PD) 30, mice were housed in groups of 3–5 mice per cage in individually ventilated polysulfone cages (Alternative design Inc., U.S.A.) within the animal rooms of the Psychology Department of National Taiwan University. Food and water were available *ad libitum*. All behavioral tests were conducted in male mice on PD 67–97, and all mice were individually housed 7 days before testing. All behavioral experiments were conducted in the dark phase. Minimal numbers of mice were used to meet the 3R reduction principle of animal use. All animal procedures were performed according to protocols approved by the Institutional Animal Care and Use Committees of National Taiwan University. Adequate measures were taken to minimize potential pain and discomfort that the mice used in this study may have experienced.

### Experiment 1: examination of growth development, protein expressions, and brain activity

Three cohorts of male mice were used in this experiment. Body weights for the 4 groups (*n* = 10/group) were measured on PD 30, 60, and 197 and used as an index for growth development. The expression levels of Akt1 and Nrg1 proteins in the cerebral cortex of adult mice (*n* = 5/group, the second cohort) were measured using Western blot with Akt1 (1:2000; Cell Signaling Technology, Inc., Danvers, MA, USA), Gapdh (1:5000; Cell Signaling Technology), and Nrg1 (1:1000; Santa Cruz Biotechnology, Inc., CA, USA) antibodies. Immune complexes were shown using appropriate peroxidase-conjugated secondary antibodies (Cell Signaling Technology). Bound antibody was detected using an enhanced chemiluminescence (ECL) kit (Millipore, Taipei, Taiwan) and densitometric analysis was performed using ImageJ software.^1^

Basic brain activity was measured in the third cohort of mice (*n* = 4/group) using micro PET-CT scans (eXplore Vista DR, GE Healthcare) with 18F-Fluorodeoxyglucose (FDG) in the Department of Nuclear Medicine of National Taiwan University Hospital, Taipei, Taiwan. The procedure and details of micro PET-CT scan were described previously (Ono et al., 2012; Chen et al., 2014). Briefly, each mouse was fasted for 15–18 h before scanning. During each scan, each subject was intraperitoneally administered 0.1 ml of FDG with an activity of 500 µCi and waited in its home cage within a sound reduced chamber for 30-min of FDG uptake. Following the uptake, each mouse was lightly anesthetized using 2–3% isoflurane and scanned for 1 h. A 6-min CT scan was conducted right after the end of 1 h PET scan to co-register the PET images with the CT images. The normalized standardized uptake values (SUV) that obtained from the target region of interest (ROI) of the mesolimbic-prefrontal loop (including the medial prefrontal cortex (mPFC) and striatum) and cerebellum (reference point) were used as an index for basic brain activity. The normalized SUV were obtained and calculated after each scan using the following formula: *SUV*_target ROI (FDG)_/*SUV*_cerebellum (FDG)_.

### Experiment 2.1: evaluation of basic behavioral phenotypes of Akt1-Nrg1 single and double mutant mice

The Experiment 2 consisted of Experiment 2.1 for basic behavioral phenotypes and Experiment 2.2 for cognitive and social functions. Basic behavioral phenotyping included open field task (for spontaneously locomotion activity), elevated plus maze task (for anxious behavior), and pre-pulse inhibition task (for sensorimotor function). For cognitive and social functions, object recognition task (for episodic-like memory), social preference and social recognition task, and delayed non-matching to sample task (DNMS; for working memory) were performed. Two cohorts of mice were used in this experiment. All behavioral tasks, except the DNMS task, were conducted in the first cohort (*n* = 10/group). Mice in the first cohort was sequentially tested with the open field task, elevated plus maze, episodic-like memory task, social preference and social recognition task, and pre-pulse inhibition with at least a 24-h interval between different tasks. The general principle of the arrangement is to avoid a more stressful task prior to a less stressful one and to minimize carryover effects. Due to food deprivation and long training period, DNMS task was performed in the second cohort of mice (*n* = 10/group). The details of behavioral tasks have been described elsewhere (Dere et al., 2005; Koike et al., 2006) or in our previous studies (Lai et al., 2006, 2014; Chen and Lai, 2011; Juan et al., 2014; Pei et al., 2014).

For basic behavioral phenotyping, open field task was used to assess the spontaneously locomotion activity. The mice were placed in a Plexiglas cage (37.5 × 21.5 × 18 cm) for 30 min observation. And the traveled distance of each mouse was recorded by EthoVision tracking system (Noldus Information Technology, the Netherlands). The elevated plus maze was used to assess the instinctively anxious behavior of novel context. The maze was elevated 50 cm from the floor and was consisted of two open arms (each 50 cm long × 10 cm wide), two closed arms with 45 cm high walls but no roof (each 50 cm long × 10 cm wide), and a square-shaped central platform (10 × 10 cm). Each mouse was placed in the central platform and faced toward one of the closed arm for 5 min observation. The time spent on each part of the maze was recorded by EthoVision tracking system (Noldus Information Technology, the Netherlands). Pre-pulse inhibition task was used as an index of sensorimotor gating function using SR-LAB startle apparatus (San Diego Instruments, San Diego, CA, USA). Under a 68 dB background noise, each session composed of 5 min accumulation period followed by 64 trials in four blocks. The pulse alone (PA) trial is a 40 ms, 120 dB white noise burst. In the pre-pulse (pp) + pulse trials, a 20 ms white noise pre-pulse stimuli of 72 dB (pp6), 78 dB (pp10), 86 dB (pp18) was presented 100 ms before a 40 ms 120 dB pulse. The non-stimulus (NS) trials presented the back ground noise only. The initial and the last blocks compose of six PA trials respectively. Two middle blocks consisted of PA, pp + pulse, and NS trials. These trials were presented pseudo-randomly and separated by inter-trial interval of 15 s on average (varying between 10 to 20 s). The percentage of pre-pulse inhibition was evaluated by the following formula: %PPI = 100 × [(PA score)−(pp-P score)]/(PA score), where the PA score was the average of the PA value in the middle blocks. The details of the testing procedure were described previously (Chen and Lai, 2011).

### Experiment 2.2: evaluation of cognitive and social functions of Akt1-Nrg1 single and double mutant mice

Three behavioral tasks were performed to evaluate cognitive and social functions in these mice, including episodic-like memory task, social preference and social recognition task, and DNMS. To simultaneously assess “what”, “where”, and “when” memory, a mouse version of episodic-like memory task was used as described previously (Dere et al., 2005). The protocol of this task was composed of habituation, sample A session, sample B session, and testing session. Each session was separated by 20 min inter-session interval. In the habituation session, the mice were placed in a Plexiglas chamber (18 cm × 18 cm × 18 cm) for 5 min. In the sample A session, mice were placed in the same chamber with four copies of a novel object A (i.e., a yellow airplane shape Lego toy) arranged in a triangle-shaped spatial configuration in the chamber for 3 min. As described previously (Dere et al., 2005), each of the 4 objects was placed in the center of the northern corner (NC), southern corner (SC), south-west corner (SW), and south-east corner (SE) of the chamber, respectively. In the sample B session, four copies of a novel object B (i.e., 10 ml glass beaker) were used to replace sample object As. The sample object Bs were placed in the center of the north-west corner (NW), north-east corner (NE), SW, and SE corners) of the chamber, respectively. Each mouse was allowed to explore the objects and chamber for 3 min. In the testing session, 2 copies of object A (“old familiar” objects placed in the NE and SW corners) and 2 copies of object B (“recent familiar” objects placed in the NW and SE corners) were used and each mouse was allowed to explore for another 3 min. The exploratory time of each object in each session was videotaped and analyzed using TopScan software (Clever Sys Inc., Reston, VA, USA). The “what”, “when” and “where” memory indexes were represented by the following formulas: what index = [Object A exploratory time/(Object A exploratory time + Object B exploratory time)] × 100; when index = [Stationary object A exploratory time/(Stationary object A exploratory time + 1/2 Object B exploratory time)] × 100; where index = [Displaced object A exploratory time/(Displaced object A exploratory time + Stationary object A exploratory time)] × 100.

To assess social behaviors, a modified version of three-chamber social preference and social recognition task was used as described previously (Pei et al., 2014). The task contained three sessions, the habituation, sociability, and social recognition sessions which were separated by 5 min inter-session intervals. In the habituation session, each mouse was placed in a Plexiglas chamber (37.5 cm × 21.5 cm × 18 cm) with two transparent plastic cylinder containers (6 cm diameter × 15 cm height) at the right and left quarter of the chamber for a 10-min habituation. In the sociability session, stranger #1 (an age and size matched (weight 27–33 g) novel WT male mouse) was placed into one of the cylinders and the other cylinder remained empty. The subject was allowed to explore the chamber for 5 min. The total sniffing time toward both cylinders was recorded and analyzed using TopScan software (Clever Sys Inc., Reston, VA, USA). The sniffing behavior was defined as the snout or the front portions of the mouse head directly touching the cylinder containers. In the social recognition session, the behavioral procedures remained the same as the sociability session, except that a second stranger (stranger #2, another age and size matched novel WT male mouse) was placed in the empty cylinder during the testing. The sociability index and social recognition index were evaluated by the following formulas: Sociability (%) = (Sniffing time toward Stranger #1/Total sniffing time) × 100; Social recognition (%) = (Sniffing time toward Stranger #2/Total sniffing time) × 100.

To assess working memory in these mice, a white acrylic T-shaped maze was used to conduct the DNMS task. Prior to the experiment, mice were food-restricted to 80–85% of their original weight. The body weight was maintained through the test period. Small pieces of food pellets were placed at two ends of the T-maze as a reward. After 2 days of habituation to the T-maze (10 min/day), 2 days of forced alternation (10 sessions/day) in the T-maze with the blocked opposite arm were conducted. In the training phase, a mouse was placed in the T-maze, forced turn to one arm, and the food reward at the terminal was consumed. After a 5-s delay, the mouse was placed in the T-maze again; to receive the food reward, the mouse was required to turn to the opposite side. When a mouse exhibited an accuracy rate >70% for three consecutive days, the testing phase was started. If not, the training phase was prolonged until the mouse fulfilled the required criteria. In the testing phase, there were 12 sessions per day and the delayed time was set to be 5, 15, and 30 s pseudorandomly. The accuracy in each delay condition was calculated as described previously (Koike et al., 2006).

### Experiment 3: examination of social interaction and social communication

Based on the findings of Experiment 2.2, social function was further examined in this experiment with another cohort of mice (*n* = 10/group). To determine social interaction and social communication in these mice, each mouse was further tested in one direct male-female encounter and a series of three indirect social encounters. The sniffing time and the number of ultra-sonic vocalization (USV) calls were used as our indexes for social interaction and social communication. In the direct encounter, each mouse was exposed to an estrous female mouse for 5 min in a novel cage, and the time spent in anogenital sniffing was recorded by a stopwatch. This encounter also served as a prior experience to facilitate USV calls later as described previously (Roullet et al., 2011). The 3-day indirect social encounter took place 7 days after the direct male-female encounter. On each testing day, each mouse sequentially encountered with another male, an ovariectomized (OVX) female, and fresh estrous female mouse urine (15 µl) in a plastic cage (25.4 × 25.4 × 40.6 cm) with a Styrofoam lid on the top for soundproofing. The novel male and OVX female were restrained in a meshed metal basket and placed in the center of the testing cage to prevent direct physical contact. The fresh female urine from an estrous female was harvested on each testing day as described previously (Roullet et al., 2011). During each test, each mouse had a 5-min clean trial and a 5-min social trial separated by a 5-min inter-trial interval. The total sniffing time was recorded by an experienced observer using a stopwatch. The sniffing behavior was defined as the snout or the front paws directly touching the meshed metal basket. For social communication, the USV emitted during each testing time was automatically recorded and analyzed using Avisoft Ultrasound Recording System and Avisoft-SASLab Pro software (Avisoft Bioacoustics, Berlin, Germany). A fast Fourier transform was conducted (512 fast Fourier transform length, 100% frame, Hamming window and 50% time window overlap). The frequency resolution was 488 Hz, time resolution was 1 ms, and cut-off frequency was 20 kHz as descripted previously (Scattoni et al., 2011). The acoustic syllable in the 30–110 kHz, 65–85 dB, 30–200 ms duration with pitch change (≦ 3 kHz) was considered as an USV call (Holy and Guo, 2005; Scattoni et al., 2011).The calls were initially auto-detected using Avisoft-SASLab Pro software and further manually checked. As a first step to investigate genotypic difference in USV calls, we simply measured total number of USV calls in Experiment 3.

### Statistical analysis

All data were normally distributed (normality test, data not shown), and the data were initially analyzed by two-way ANOVA for gene-gene interaction and main effect of each gene. *Post hoc* analysis was performed with Fisher’s LSD test when *F* values reached significant difference. And two-tailed *t*-test was used for the social behaviors. Statistical analysis was performed by SPSS18. Alpha was set at 0.05 and adjusted *p* values < 0.05 were considered statistically significant.

## Results

### Experiment 1: examination of general features

**Figure 1:**
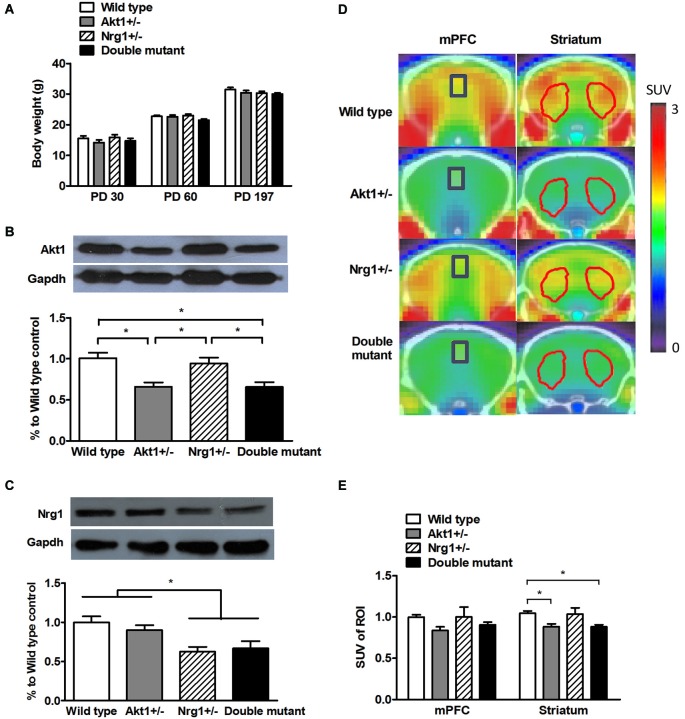
**General features of wild-type (WT), *Akt1^+/−^*, *Nrg1^+/−^*, and double mutant mice in Experiment 1. (A)** Physical growth at postnatal day (PD) 30, 60, and 197. **(B)** Expression of Akt1 protein levels in the cerebral cortices of the 4 groups of mice. A ~30–40% reduction of Akt1 protein was found in both Akt1^+/−^ and double mutant mice. **(C)** The expression of Nrg1 protein level in the cerebral cortices of the 4 groups of mice. A ~40–45% reduction of Nrg1 protein was found in both Nrg1^+/−^ and double mutant mice. **(D)** Representative PET-CT fusion images of coronal slices of the 4 groups of mice using microPET with ^18^F-FDG. The medial prefrontal cortices (mPFC, Bregma 1.96 mm) are highlighted in blue rectangles, and the striatums (Bregma 0.5 mm) are highlighted in red polygons. **(E)** The normalized and averaged standardized uptake values (SUV) in the brain regions of interest (ROI, including the mPFC and striatum) among the 4 groups. The ratio was calculated as *SUV_target ROI (FDG)_*/SUV_cerebellum (FDG)_. *p < 0.05.

As depicted in Figure 1A, no significant genotypic difference was found in body weight on PD 30 (interaction: *F*_(1,56)_ = 0.02, *p* = 0.93; *Akt1* main effect: *F*_(1,56)_ = 2.34, *p* = 0.13; *Nrg1* main effect: *F*_(1,56)_ = 0.37, *p* = 0.54), PD 60 (interaction: *F*_(1,56)_ = 2.16, *p* = 0.15; *Akt1* main effect: *F*_(1,56)_ = 3.23, *p* = 0.78; *Nrg1* main effect: *F*_(1,56)_ = 0.72, *p* = 0.40), and PD 197 (interaction: *F*_(1,56)_ = 0.36, *p* = 0.55; *Akt1* main effect: *F*_(1,56)_ = 1.07, *p* = 0.31; *Nrg1* main effect: *F*_(1,56)_ = 1.60, *p* = 0.21) indicating normal growth and development. As shown in Figure 1B, a significant gene dose effect of Akt1 expression was found across the 4 groups as expected (interaction: *F*_(1,16)_ = 0.24, *p* = 0.63; *Akt1* main effect: *F*_(1,16)_ = 25.10, *p* < 0.05; *post hoc* comparison all *p* < 0.05). An expected result was also found in Nrg1 expression as depicted in Figure 1C (interaction: *F*_(1,16)_ = 0.93, *p* = 0.35; *Nrg1* main effect: *F*_(1,16)_ = 16.84, *p* < 0.05; *post hoc* comparison all *p* < 0.05). Representative PET scan images are shown in Figure 1D and PET scan images revealed no genotypic interaction in the mPFC (*F*_(1,12)_ = 0.21, *p* = 0.66) and striatum (*F*_(1,12)_ = 0.16, *p* = 0.70). But a significant main effect of Akt1 was found in the striatum (*F*_(1,12)_ = 11.00, *p* < 0.05). As depicted in Figure 1E, both *Akt1*-deficient mice and double mutant mice had less striatal glucose uptake compared to their WT controls (*post hoc* comparison *p* < 0.05), indicating less brain activity in the striatum. No significant difference was found between these two groups.

### Experiment 2.1: basic behavioral phenotyping

As indicated in Table 1 (top half), our basic behavioral phenotyping revealed that there were no significant genotypic interaction and main effects in locomotor activity (interaction: *F*_(1,36)_ = 2.98, *p* = 0.09; *Akt1* main effect: *F*_(1,36)_ = 0.01, *p* = 0.92; *Nrg1* main effect: *F*_(1,36)_ = 0.52, *p* = 0.48), and anxiety-like behavior (interaction: *F*_(1,36)_ = 0.29, *p* = 0.59; *Akt1* main effect: *F*_(1,36)_ = 0.75, *p* = 0.39; *Nrg1* main effect: *F*_(1,36)_ = 0.13, *p* = 0.72) and sensorimotor gating function at PP6 (interaction: *F*_(1,36)_ = 0.77, *p* = 0.39; *Akt1* main effect: *F*_(1,36)_ = 4.25, *p* = 0.06; *Nrg1* main effect: F_(1,36)_ = 0.41, *p* = 0.52), PP10 (interaction: *F*_(1,36)_ = 2.02, *p* = 0.16; *Akt1* main effect: *F*_(1,36)_ = 0.99, *p* = 0.33; *Nrg1* main effect: *F*_(1,36)_ = 1.55, *p* = 0.22) and PP18 (interaction: *F*_(1,36)_ = 1.12, *p* = 0.30; *Akt1* main effect: *F*_(1,36)_ = 0.52, *p* = 0.48; *Nrg1* main effect: *F*_(1,36)_ = 0.13, *p* = 0.72).

**Table 1:** **A summary table of behavioral results (means ± SEM) for wild-type, *Akt1*^+/−^, *Nrg1*^+/−^, and double mutant mice (*n* = 10/group) in Experiments 2.1 and 2.2 (except sociability and social recognition task)**.

Behavioral task	Wild type	*Akt1*^+/−^	*Nrg1*^+/−^	Double mutant
**Open field**
Travel distance (cm)	10332.40 ± 372.61	9516.88 ± 509.15	9882.61 ± 455.20	10612.40 ± 443.08
**Elevated plus maze**
Time spent in open arm (%)	20.56 ± 3.63	15.95 ± 3.58	17.61 ± 3.64	16.73 ± 2.08
**Pre-pulse inhibition**				
PP6 (72 dB, %)	31.14 ± 4.14	50.03 ± 6.07	41.29 ± 6.47	48.60 ± 8.11
PP10 (78 dB, %)	39.17 ± 5.18	54.41 ± 5.24	55.93 ± 6.92	53.36 ± 6.47
PP18 (86 dB, %)	62.82 ± 4.13	73.66 ± 4.45	67.12 ± 8.24	65.05 ± 6.59
**Working memory**				
Accuracy (delay period, %)				
5 s	82.50 ± 1.95	83.33 ± 2.78	80.80 ± 3.74	83.34 ± 3.51
15 s	76.67 ± 3.8	71.67 ± 4.34	71.67 ± 4.16	74.17 ± 3.39
30 s	72.50 ± 3.74	72.50 ± 4.31	65.00 ± 4.44	65.01 ± 3.24
**Episodic-like memory**				
Time of exploration (s)				
*What discrimination*
(object A vs. object B)	71.45 ± 3.40	74.94 ± 4.95	65.16 ± 3.48	67.50 ± 2.89
*When discrimination*
(stationary A vs. object B/2)	58.58 ± 3.94	65.21 ± 5.73	60.43 ± 5.39	64.09 ± 4.06
*Where discrimination*
(displaced A vs. stationary A)	70.67 ± 2.99	66.25 ± 4.09	53.38 ± 6.73*	53.93 ± 5.52*

Two cohorts (4 groups, *n* = 10 each) of mice were used. All behavioral tasks, except delayed non-match to sample (DNMS) task, were conducted in the first cohort. Mice in the first cohort was sequentially tested with the open field task, elevated plus maze, episodic-like memory task (i.e., what, when and where memories), social preference and social recognition task, and pre-pulse inhibition with at least a 24-h interval between different tasks. The DNMS task was conducted in the second cohort of mice due to food deprivation and long training period. * *p* < 0.05 compared to wild-type and Akt1^+/−^ mice.

### Experiment 2.2: evaluation of cognitive and social functions

As indicated in Table 1 (bottom half), in the episodic-like memory task, no significant genotypic interaction and main effects were found in the “what” memory (interaction: *F*_(1,36)_ = 0.02, *p* = 0.88; *Akt1* main effect: *F*_(1,36)_ = 0.60, *p* = 0.44; *Nrg1* main effect: *F*_(1,36)_ = 3.33, *p* = 0.08), and “when” memory (interaction: *F*_(1,36)_ = 0.10, *p* = 0.76; *Akt1* main effect: *F*_(1,36)_ = 1.13, *p* = 0.30; *Nrg1* main effect: *F*_(1,36)_ = 0.01, *p* = 0.94). However, there is a main effect of *Nrg1* genotype in “where” memory (interaction: *F*_(1,36)_ = 0.24, *p* = 0.63; *Akt1* main effect: *F*_(1,36)_ = 0.15, *p* = 0.70; *Nrg1* main effect: *F*_(1,36)_ = 8.64, *p* < 0.05); *Nrg1*^+/−^ and double mutant mice showed significantly lower ability to discriminate the spatial trace compared to *Akt1*^+/−^ and wild type groups (*post hoc* all *p* < 0.05).

**Figure 2:**
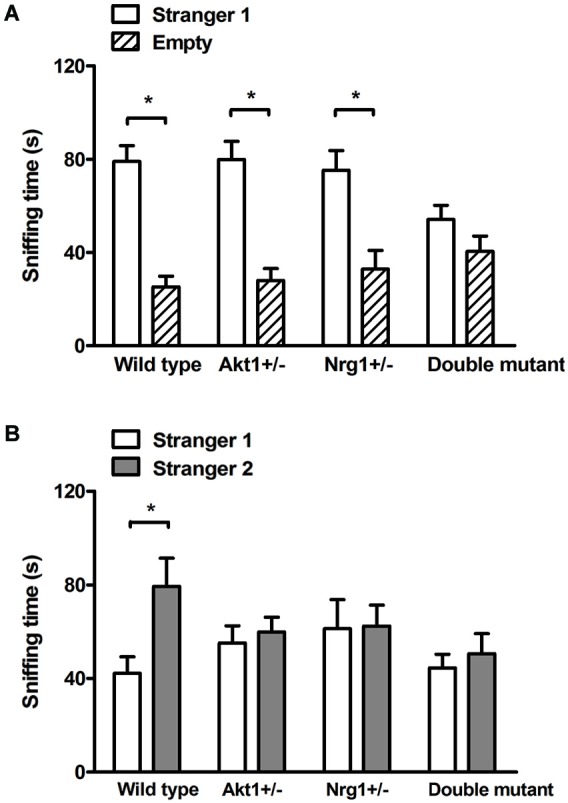
**Total sniffing time (means ± SEM sec) of WT, *Akt1^+/−^*, *Nrg1*^+/−^, and double mutant mice in the sociability and social recognition task of Experiment 2.2. (A)** In the 5-min sociability test, all mice (except double mutant mice) displayed a significant social preference toward the stimulus (i.e., stranger 1) mouse. **(B)** In the 5-min social recognition test (5-min after the end of the sociability test), only WT mice displayed a significant preference toward a novel (i.e., stranger 2) mouse. *p < 0.05.

In the sociability test, no genotypic interaction was found (*F*_(1,36)_ = 3.84, *p* = 0.58) but there were significant main effects on *Akt1* genotype (*F*_(1,36)_ = 7.33, *p* < 0.05) and *Nrg1* genotype (*F*_(1,36)_ = 9.93, *p* < 0.05). As shown in Figure 2A, WT, *Akt1*^+/−^, and *Nrg1*^+/−^ mice spent significantly more time on sniffing Stranger 1 compared to the empty cylinder (*t*_(9)_ = 11.09, *p* < 0.05; *t*_(9)_ = 9.10, *p* < 0.05; *t*_(9)_ = 8.42, *p* < 0.05, respectively). A trend but not significant difference was found in double mutant mice (*t*_(9)_ = 2.25, *p* = 0.051). In the social recognition test, no genotypic interaction was found (*F*_(1,36)_ = 3.89, *p* = 0.06) but there were significant main effects on *Akt1* genotype (*F*_(1,36)_ = 6.42, *p* < 0.05) and Nrg1 genotype (*F*_(1,36)_ = 5.65, *p* < 0.05). As shown in Figure 2B, WT mice displayed a significant preference toward a novel mouse (*t*_(9)_ = −4.86, *p* < 0.05). In contrast, *Akt1*^+/−^, *Nrg1*^+/−^, and double mutant mice did not show such preference (all *p* > 0.05).

Also as indicated in Table 1, no significant genotypic interaction and main effects were found in the DNMS task at 5s delay period (interaction: *F*_(1,36)_ = 0.07, *p* = 0.78; *Akt1* main effect: *F*_(1,36)_ = 0.29, *p* = 0.59; *Nrg1* main effect: *F*_(1,36)_ = 0.07, *p* = 0.78), 15s delay period (interaction: *F*_(1,36)_ = 0.90, *p* = 0.35; *Akt1* main effect: *F*_(1,36)_ = 0.10, *p* = 0.75; *Nrg1* main effect: *F*_(1,36)_ = 0.10, *p* = 0.75) and 30 s delay period (interaction: *F*_(1,36)_ = 0.00, *p* = 1.00; *Akt1* main effect: *F*_(1,36)_ = 0.00, *p* = 1.00; *Nrg1* main effect: *F*_(1,36)_ = 3.58, *p* = 0.07), respectively.

### Experiment 3: social interaction and social communication

**Figure 3:**
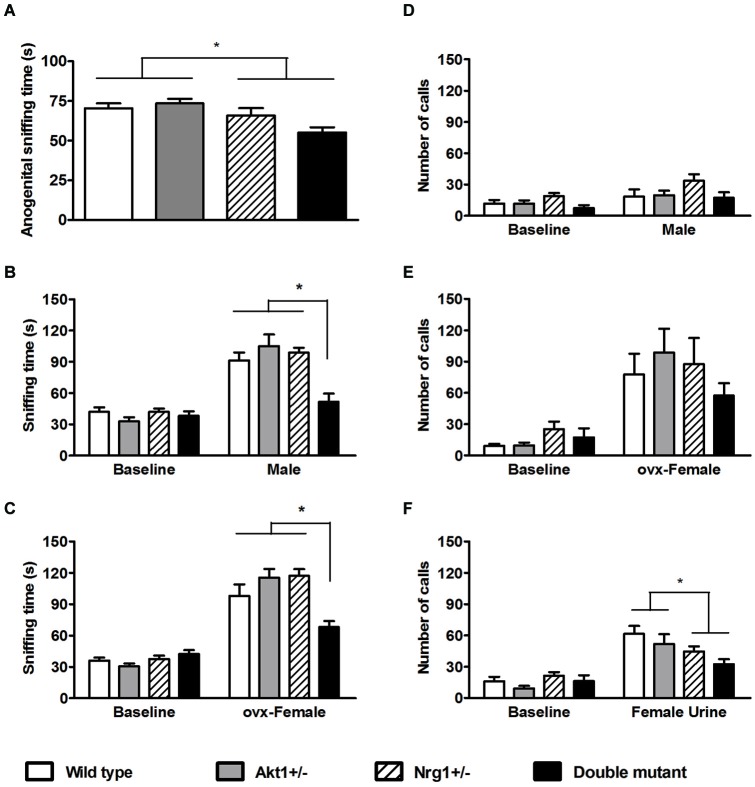
**Total sniffing time and number of calls (means ± SEM) of WT, *Akt1^+/−^*, *Nrg1*^+/−^, and double mutant mice in the social interaction and social communication task in Experiment 3. (A)** The total anogenital sniffing time during a 5-min direct encounter with an estrous female mouse. **(B,C)** The total sniffing time during a 5-min non-stimulus baseline and a 5-min indirect social encounter with another male or an ovariectomized (OVX) female, respectively. **(D,E,F)** Total number of ultra-sonic vocalization (USV) calls during a 5-min non-stimulus baseline and a 5-min indirect social encounter with another male, an OVX female, and 15 µl of fresh female urine, respectively. *p < 0.05.

For the anogenital sniffing time in the direct male-female encounter, no significant genotypic interaction was found (*F*_(1,36)_ = 3.43, *p* = 0.07). But as depicted in Figure 3A, there was a main effect on Nrg1 genotype (*F*_(1,36)_ = 11.50, *p* < 0.05). In the indirect social encounters, a genotypic interaction was found in the novel male encounter (*F*_(1,36)_ = 6.20, *p* < 0.05). As shown in Figure 3B, statistical analysis further revealed significant simple main effects of Akt1^+/−^ at Nrg1^+/−^ condition (*F*_(1,36)_ = 14.91, *p* < 0.05) and Nrg1^+/−^ at Akt1^+/−^ condition (*F*_(1,36)_ = 19.14, *p* < 0.05), indicating a significantly synergistic reduction of sniffing time in the double mutant mice. In OVX female encounter, a genotypic interaction was also found (*F*_(1,36)_ = 16.67, *p* < 0.05). As shown in Figure 3C, statistical analysis further revealed significant simple main effects of Akt1^+/−^ at Nrg1^+/−^ condition (*F*_(1,36)_ = 18.10, *p* < 0.05) and of Nrg1^+/−^ at Akt1^+/−^ condition (*F*_(1,36)_ = 16.94, *p* < 0.05), indicating a significantly synergistic reduction of sniffing time in the double mutant mice as well.

In the number of USV calls during the 3 indirect social encounters, the presentation of a novel male did not increase total number of calls and no significant difference among the 4 groups was found (Figure 3D, interaction: *F*_(1,36)_ = 2.30, *p* = 0.14; *Akt1* main effect: *F*_(1,36)_ = 1.71, *p* = 0.20; *Nrg1* main effect: *F*_(1, 36)_ = 1.21, *p* = 0.28). The presentation of OVX females significantly induced higher number of total USV calls, but no significant genotypic interaction or main effects were found among the 4 groups (Figure 3E, interaction: *F*_(1,36)_ = 2.05, *p* = 0.16; *Akt1* main effect: *F*_(1,36)_ = 0.00, *p* = 0.99; *Nrg1* main effect: *F*_(1,36)_ = 1.07, *p* = 0.31). In contrast, the use of fresh estrous female urine significantly increased the total number of USV calls. As depicted in Figure 3F, there was a significant main effect of *Nrg1* genotype (*F*_(1,36)_ = 6.89, *p* < 0.05) and *Nrg1*-deficient mice emitted a fewer number of USV calls compared to WT and *Akt1*^+/−^ mice. Furthermore, it was reported that the 22- and 55-kHz calls are two classic USV calls which reflect the presence of the aversive motivational state and positively appetitive motivation in adult rats, respectively (Brudzynski, 2007). We further analyzed our USV data and found that most of the USV calls recorded in the 4 groups of mice in this experiment are above 55 kHz. Only a few 22-kHz calls were identified in our mice during their encounters with an OVX female and the female mouse urine. Although the frequency range of USV calls in male mice are between 30–110 kHz while they encounter with female mice or female urinary pheromone, the functional significance of different USV syllables are still unknown (Holy and Guo, 2005). Thus, compared to positive and negative calls in rats, the total number of USV calls can be used as an index for social communication in our mice in the present experiment.

## Discussion

In this study, *Akt1-Nrg1* single and double mutant mice were produced and used to characterize behavioral phenotypes and cognitive/social functions of these mice. The amount of each genotype produced from our breeding pairs appears to follow Mendelian frequency in both sexes (data not shown), indicating that partial deficiency of these genes/proteins is not lethal. In Experiment 1, we found that the general development and brain activity in the mPFC of these mutant mice are similar to their WT controls. The expression of Akt1/Nrg1 proteins in the cerebral cortices of double mutant mice occurred in a gene-dosage-dependent manner as reported previously in single mutant mice (Chen et al., 2012; Pei et al., 2014). In Experiment 2, our behavioral phenotyping revealed that mutant mice have a normal behavioral profile except for their episodic-like memory and social function (especially in double mutant mice). In Experiment 3, a synergistic effect of *Akt1* and *Nrg1* was further confirmed in double mutant mice in that they had impaired social interaction compared to the other 3 groups. To the best of our knowledge, this is the first study to examine behavioral consequence of *Akt1* and *Nrg1* double deficiency in mice, and our data revealed a synergistic epistasis effect of these two genes, especially in social interaction.

Either single or double deficiency of these two schizophrenia-related candidate genes appeared to have no significant effect on general behavioral functions and basic brain activity in the mPFC. It was proposed that dopaminergic pathways play a vital role in the dopamine hypothesis of schizophrenia (Howes and Kapur, 2009) and that the dysregulation of prefrontal-temporolimbic cortical pathways might be involved in the prevalence of schizophrenia-related pathology (Carlsson et al., 2001; Laruelle et al., 2003). Our FDG-PET scan indicated that the deficiency of either *Akt1* or both genes might marginally affect basic glucose metabolism in the mPFC and striatum, two critical dopaminergic areas in the brain, at least at the baseline level. In contrast, *Nrg1* appears to have an independent effect in this case. Our general behavioral phenotyping revealed that the deficiency of either or both genes did not result in impairment of basic behavioral functions, including spontaneous locomotor activity, anxiety-like behavior, sensorimotor gating function, and working memory. These findings are consistent or similar with those behavioral phenotyping findings in *Akt1* homozygous mutant mice (Lai et al., 2006; Chen and Lai, 2011) and two strains of *Nrg1* TM-domain mutant mice (O’Tuathaigh et al., 2007; Pei et al., 2014). In contrast, our *Nrg1*^+/−^ and double mutant mice displayed impaired “where” memory in the episodic-like memory testing. Similarly, healthy human subjects that carry *NRG1* risk alleles at rs35753505 were reported to display hyperactivation in the episodic memory-related brain areas using fMRI (Krug et al., 2010).

Importantly, a synergistic action of *Akt1* and *Nrg1* genes was found in the social interaction of our double mutant mice. Positive epistasis is usually defined as the phenotype is higher than expected and negative epistasis is defined as the phenotype is lower than expected (Phillips, 2008; Lehner, 2011). In our study, double mutant mice exhibited less social preference toward stimulus males in the sociability test, suggesting a negative epistasis. A negative epistatic interaction between *Akt1* and *Nrg1* on social interaction was further confirmed in our double mutant mice, especially during their indirect social encounters with a male and an OVX female. One possible explanation for the negative epistatic interaction between *Akt1* and *Nrg1* in our mutant mice might be resulted from a potential hyperactivation of glycogen synthase kinase-3 (GSK3, a key downstream kinase of Akt1). In a similar vein, hyperactivation of GSK3 was reported in *fragile X mental retardation 1* knock-out mice which might contribute to social recognition deficit and could be ameliorated by chronic lithium (GSK3 inhibitor) treatment (Mines et al., 2010). Thus, these studies support the importance of Nrg1-Akt1 signaling pathway in sociability and social interaction and also suggest an epistatic but functional redundancy role of *Nrg1* and *Akt1* in the modulation of social functions. As indicated in the social preference task of Experiment 2 and the male-male social interaction of Experiment 3, the deficiency of either gene in our mice appeared to have less or no effect on their social preference, suggesting a functional redundancy role of *Nrg1* and *Akt1* in sociability. In contrast, we found that the deficiency of either gene or both genes might affect their social recognition function compared to their WT littermates, suggesting a necessary role for *Nrg1* and *Akt1* in the social recognition. Furthermore, an impairment of social recognition and reductions of hippocampal GAD67 and parvalbumin expressions were also reported in *Nrg1*^+/−^ male mice (Pei et al., 2014), which is consistent with our current behavioral finding and suggests the vulnerability of hippocampal GABAergic system in these mutant mice. It should be noted that a series of control experiments regarding normal profiles of basic behavioral phenotypes was reported previously in *Akt1*^+/−^ or *Akt1^−/–^* mice (Lai et al., 2006; Chen and Lai, 2011; Chen et al., 2012, 2014) and in *Nrg1*^+/−^ mice (Pei et al., 2014), such as locomotor activity, anxiety-like behavior, olfactory function, and sensorimotor gating function. Both *Akt1*^+/−^ and *Nrg1*^+/−^ male mice also displayed preference toward the higher concentration of sucrose solution (Chen et al., 2012; Pei et al., 2014), indicating that they have normal preference for sucrose and motivation for reward. Although we did not examine biochemical alterations in the brains of these mice in the present study, it is plausible that a lower level of Nrg1-stimulated Akt phosphorylation and down regulation of PI3-kinase/AKT signaling (or hyperactivation of GSK3) might play an important role in these observed behavioral impairments, as has been suggested in other studies (Keri et al., 2009a,b; Seres et al., 2010).

Interestingly, our current findings in mutant mice can be further compared with human imaging studies in which epistatic interactions between AKT1 and NRG1 have been previously investigated. For example, it was reported that healthy human subjects that carry NRG1 risk alleles (rs10503929) and AKT1 risk alleles (rs2494734) displayed normal activity in the dorsolateral prefrontal cortex (DLPFC) using fMRI whereas the subjects carry NRG1(rs10503929), ERBB4 (rs1026882) and AKT1 (rs2494734) displayed higher activity in DLPFC (Nicodemus et al., 2010). This result is somewhat support by our mouse data in the DNMS task for working memory. Besides, B lymphoblast of schizophrenic patients with COMT Val/Val and NRG243177 T/T displayed poorer Nrg1-induced cell migration and adhesion (Sei et al., 2007). And lower AKT1 phosphorylation was found in COMT Met/Met carriers with AKT1 rs1130233 G/A risk allele (Sei et al., 2010). It is possible that the poorer Nrg1-induced cell migration and adhesion in schizophrenic patients might be resulted from AKT1 and NRG1 risk alleles. Likewise, the impairment of sociability in our double mutant mice could be caused by both AKT1 and NRG1 deficiencies as reported in schizophrenic patients. It is of interest to further investigate the relationship between social behaviors and neural development in Akt1, Nrg1, and Akt1-Nrg1 mutant mice.

Collectively, the use of double mutant mice provides a feasible and complementary model to study epistasis effects of genes in the pathogenesis of schizophrenia-related behavioral symptoms. Our findings indicate that the deficiency of *Akt1* and *Nrg1* have negative epistatic effect on the social functions, which is worth further investigation.

## Conflict of interest statement

The authors declare that the research was conducted in the absence of any commercial or financial relationships that could be construed as a potential conflict of interest.

## Footnotes

^1^http://imagej.nih.gov/ij/

## References

[B1] Arguello P. A. Gogos J. A. (2006). Modeling madness in mice: one piece at a time. Neuron 52, 179–196. 10.1016/j.neuron.2006.09.023 17015235

[B2] Bao J. Wolpowitz D. Role L. W. Talmage D. A. (2003). Back signaling by the Nrg-1 intracellular domain. J. Cell Biol. 161, 1133–1141. 10.1083/jcb.200212085 12821646 PMC2172983

[B3] Brudzynski S. M. (2007). Ultrasonic calls of rats as indicator variables of negative or positive states: acetylcholine-dopamine interaction and acoustic coding. Behav. Brain Res. 182, 261–273. 10.1016/j.bbr.2007.03.004 17467067

[B4] Carlsson A. Waters N. Holm-Waters S. Tedroff J. Nilsson M. Carlsson M. L. (2001). Interactions between monoamines, glutamate and GABA in schizophrenia: new evidence. Annu. Rev. Pharmacol. Toxicol. 41, 237–260. 10.1146/annurev.pharmtox.41.1.237 11264457

[B5] Chen Y. C. Chen Y. W. Hsu Y. F. Chang W. T. Hsiao C. K. Min M. Y.. (2012). Akt1 deficiency modulates reward learning and reward prediction error in mice. Genes Brain Behav. 11, 157–169. 10.1111/j.1601-183x.2011.00759.x 22151747

[B6] Chen Y. W. Kao H. Y. Min M. Y. Lai W. S. (2014). A sex- and region-specific role of Akt1 in the modulation of methamphetamine-induced hyperlocomotion and striatal neuronal activity: implications in schizophrenia and methamphetamine-induced psychosis. Schizophr. Bull. 40, 388–398. 10.1093/schbul/sbt031 23474853 PMC3932084

[B7] Chen Y. W. Lai W. S. (2011). Behavioral phenotyping of v-akt murine thymoma viral oncogene homolog 1-deficient mice reveals a sex-specific prepulse inhibition deficit in females that can be partially alleviated by glycogen synthase kinase-3 inhibitors but not by antipsychotics. Neuroscience 174, 178–189. 10.1016/j.neuroscience.2010.09.056 20888398

[B8] Cho H. Mu J. Kim J. K. Thorvaldsen J. L. Chu Q. Crenshaw E. B.. (2001). Insulin resistance and a diabetes mellitus-like syndrome in mice lacking the protein kinase Akt2 (PKB beta). Science 292, 1728–1731. 10.1126/science.292.5522.1728 11387480

[B9] Dere E. Huston J. P. De Souza Silva M. A. (2005). Episodic-like memory in mice: simultaneous assessment of object, place and temporal order memory. Brain Res. Brain Res. Protoc. 16, 10–19. 10.1016/j.brainresprot.2005.08.001 16185914

[B10] Downward J. (1998). Mechanisms and consequences of activation of protein kinase B/Akt. Curr. Opin. Cell Biol. 10, 262–267. 10.1016/s0955-0674(98)80149-x 9561851

[B11] Elvevåg B. Goldberg T. E. (2000). Cognitive impairment in schizophrenia is the core of the disorder. Crit. Rev. Neurobiol. 14, 1–21. 10.1615/critrevneurobiol.v14.i1.10 11253953

[B12] Emamian E. S. (2012). AKT/GSK3 signaling pathway and schizophrenia. Front. Mol. Neurosci. 5:33. 10.3389/fnmol.2012.00033 22435049 PMC3304298

[B13] Emamian E. S. Hall D. Birnbaum M. J. Karayiorgou M. Gogos J. A. (2004). Convergent evidence for impaired AKT1-GSK3beta signaling in schizophrenia. Nat. Genet. 36, 131–137. 10.1038/ng1296 14745448

[B14] Falls D. L. (2003). Neuregulins: functions, forms and signaling strategies. Exp. Cell Res. 284, 14–30. 10.1016/s0014-4827(02)00102-7 12648463

[B15] Green M. F. (2006). Cognitive impairment and functional outcome in schizophrenia and bipolar disorder. J. Clin. Psychiatry 67(Suppl. 9), 3–8; discussion 36–42. 10.4088/JCP.1006e12 16965182

[B16] Harrison P. J. Law A. J. (2006). Neuregulin 1 and schizophrenia: genetics, gene expression and neurobiology. Biol. Psychiatry 60, 132–140. 10.1016/j.biopsych.2005.11.002 16442083

[B17] Harrison P. J. Weinberger D. R. (2005). Schizophrenia genes, gene expression and neuropathology: on the matter of their convergence. Mol. Psychiatry 10, 40–68. 10.1038/sj.mp.4001558 15263907

[B18] Holy T. E. Guo Z. (2005). Ultrasonic songs of male mice. PLoS Biol. 3:e386. 10.1371/journal.pbio.0030386 16248680 PMC1275525

[B19] Howes O. D. Kapur S. (2009). The dopamine hypothesis of schizophrenia: version III–the final common pathway. Schizophr. Bull. 35, 549–562. 10.1093/schbul/sbp006 19325164 PMC2669582

[B20] Juan L. W. Liao C. C. Lai W. S. Chang C. Y. Pei J. C. Wong W. R.. (2014). Phenotypic characterization of C57BL/6J mice carrying the Disc1 gene from the 129S6/SvEv strain. Brain Struct. Funct. 219, 1417–1431. 10.1007/s00429-013-0577-8 23689501

[B21] Kanakry C. G. Li Z. Nakai Y. Sei Y. Weinberger D. R. (2007). Neuregulin-1 regulates cell adhesion via an ErbB2/phosphoinositide-3 kinase/Akt-dependent pathway: potential implications for schizophrenia and cancer. PLoS One 2:e1369. 10.1371/journal.pone.0001369 18159252 PMC2147048

[B22] Keri S. Beniczky S. Kelemen O. (2010). Suppression of the P50 evoked response and neuregulin 1-induced AKT phosphorylation in first-episode schizophrenia. Am. J. Psychiatry 167, 444–450. 10.1176/appi.ajp.2009.09050723 20048019

[B23] Keri S. Seres I. Kelemen O. Benedek G. (2009a). Neuregulin 1-stimulated phosphorylation of AKT in psychotic disorders and its relationship with neurocognitive functions. Neurochem. Int. 55, 606–609. 10.1016/j.neuint.2009.06.002 19524002

[B24] Keri S. Seres I. Kelemen O. Benedek G. (2009b). The relationship among neuregulin 1-stimulated phosphorylation of AKT, psychosis proneness and habituation of arousal in nonclinical individuals. Schizophr. Bull. 37, 141–147. 10.1093/schbul/sbp063 19549627 PMC3004188

[B25] Koike H. Arguello P. A. Kvajo M. Karayiorgou M. Gogos J. A. (2006). Disc1 is mutated in the 129S6/SvEv strain and modulates working memory in mice. Proc. Natl. Acad. Sci. U S A 103, 3693–3697. 10.1073/pnas.0511189103 16484369 PMC1450143

[B26] Krishnan V. Han M. H. Mazei-Robison M. Iniguez S. D. Ables J. L. Vialou V.. (2008). AKT signaling within the ventral tegmental area regulates cellular and behavioral responses to stressful stimuli. Biol. Psychiatry 64, 691–700. 10.1016/j.biopsych.2008.06.003 18639865 PMC2742561

[B27] Krug A. Markov V. Krach S. Jansen A. Zerres K. Eggermann T.. (2010). The effect of Neuregulin 1 on neural correlates of episodic memory encoding and retrieval. Neuroimage 53, 985–991. 10.1016/j.neuroimage.2009.12.062 20036336

[B28] Kvajo M. Mckellar H. Gogos J. A. (2010). Molecules, signaling and schizophrenia. Curr. Top. Behav. Neurosci. 4, 629–656. 10.1007/7854_2010_41 21312416

[B29] Lai W. S. Chang C. Y. Wong W. R. Pei J. C. Chen Y. S. Hung W. L. (2014). Assessing schizophrenia-relevant cognitive and social deficits in mice: a selection of mouse behavioral tasks and potential therapeutic compounds. Curr. Pharm. Des. 20, 5139–5150. 10.2174/1381612819666140110122750 24410559

[B30] Lai W. S. Xu B. Westphal K. G. Paterlini M. Olivier B. Pavlidis P.. (2006). Akt1 deficiency affects neuronal morphology and predisposes to abnormalities in prefrontal cortex functioning. Proc. Natl. Acad. Sci. U S A 103, 16906–16911. 10.1073/pnas.0604994103 17077150 PMC1636552

[B31] Laruelle M. Kegeles L. S. Abi-Dargham A. (2003). Glutamate, dopamine and schizophrenia: from pathophysiology to treatment. Ann. N Y Acad. Sci. 1003, 138–158. 10.1196/annals.1300.063 14684442

[B32] Lehner B. (2011). Molecular mechanisms of epistasis within and between genes. Trends Genet. 27, 323–331. 10.1016/j.tig.2011.05.007 21684621

[B33] Marmor M. D. Skaria K. B. Yarden Y. (2004). Signal transduction and oncogenesis by ErbB/HER receptors. Int. J. Radiat. Oncol. Biol. Phys. 58, 903–913. 10.1016/j.ijrobp.2003.06.002 14967450

[B34] Mei L. Xiong W. C. (2008). Neuregulin 1 in neural development, synaptic plasticity and schizophrenia. Nat. Rev. Neurosci. 9, 437–452. 10.1038/nrn2392 18478032 PMC2682371

[B35] Mines M. A. Yuskaitis C. J. King M. K. Beurel E. Jope R. S. (2010). GSK3 influences social preference and anxiety-related behaviors during social interaction in a mouse model of fragile X syndrome and autism. PLoS One 5:e9706. 10.1371/journal.pone.0009706 20300527 PMC2838793

[B36] Nicodemus K. K. Law A. J. Radulescu E. Luna A. Kolachana B. Vakkalanka R.. (2010). Biological validation of increased schizophrenia risk with NRG1, ERBB4 and AKT1 epistasis via functional neuroimaging in healthy controls. Arch. Gen. Psychiatry 67, 991–1001. 10.1001/archgenpsychiatry.2010.117 20921115 PMC4291187

[B37] Norton N. Williams H. J. Owen M. J. (2006). An update on the genetics of schizophrenia. Curr. Opin. Psychiatry 19, 158–164. 10.1097/01.yco.0000214341.52249.59 16612196

[B38] Ono Y. Lin H. C. Tzen K. Y. Chen H. H. Yang P. F. Lai W. S.. (2012). Active coping with stress suppresses glucose metabolism in the rat hypothalamus. Stress 15, 207–217. 10.3109/10253890.2011.614296 21936685

[B39] O’Tuathaigh C. M. Babovic D. O’Sullivan G. J. Clifford J. J. Tighe O. Croke D. T.. (2007). Phenotypic characterization of spatial cognition and social behavior in mice with ‘knockout’ of the schizophrenia risk gene neuregulin 1. Neuroscience 147, 18–27. 10.1016/j.neuroscience.2007.03.051 17512671

[B40] O’Tuathaigh C. M. O’Connor A. M. O’Sullivan G. J. Lai D. Harvey R. Croke D. T.. (2008). Disruption to social dyadic interactions but not emotional/anxiety-related behaviour in mice with heterozygous ‘knockout’ of the schizophrenia risk gene neuregulin-1. Prog. Neuropsychopharmacol. Biol. Psychiatry 32, 462–466. 10.1016/j.pnpbp.2007.09.018 17980471

[B41] Pei J. C. Liu C. M. Lai W. S. (2014). Distinct phenotypes of new transmembrane-domain neuregulin 1 mutant mice and the rescue effects of valproate on the observed schizophrenia-related cognitive deficits. Front. Behav. Neurosci. 8:126. 10.3389/fnbeh.2014.00126 24782733 PMC3995064

[B42] Phillips P. C. (2008). Epistasis–the essential role of gene interactions in the structure and evolution of genetic systems. Nat. Rev. Genet. 9, 855–867. 10.1038/nrg2452 18852697 PMC2689140

[B43] Roullet F. I. Wohr M. Crawley J. N. (2011). Female urine-induced male mice ultrasonic vocalizations, but not scent-marking, is modulated by social experience. Behav. Brain Res. 216, 19–28. 10.1016/j.bbr.2010.06.004 20540967 PMC3094925

[B44] Scattoni M. L. Ricceri L. Crawley J. N. (2011). Unusual repertoire of vocalizations in adult BTBR T+tf/J mice during three types of social encounters. Genes Brain Behav. 10, 44–56. 10.1111/j.1601-183x.2010.00623.x 20618443 PMC2972364

[B45] Schwab S. G. Wildenauer D. B. (2009). Update on key previously proposed candidate genes for schizophrenia. Curr. Opin. Psychiatry 22, 147–153. 10.1097/YCO.0b013e328325a598 19553868

[B46] Sei Y. Li Z. Song J. Ren-Patterson R. Tunbridge E. M. Iizuka Y.. (2010). Epistatic and functional interactions of catechol-o-methyltransferase (COMT) and AKT1 on neuregulin1-ErbB signaling in cell models. PLoS One 5:e10789. 10.1371/journal.pone.0010789 20520724 PMC2875391

[B47] Sei Y. Ren-Patterson R. Li Z. Tunbridge E. M. Egan M. F. Kolachana B. S.. (2007). Neuregulin1-induced cell migration is impaired in schizophrenia: association with neuregulin1 and catechol-o-methyltransferase gene polymorphisms. Mol. Psychiatry 12, 946–957. 10.1038/sj.mp.4001994 17440436

[B48] Seres I. Kelemen O. Benedek G. Kéri S. (2010). Neuregulin 1-induced AKT phosphorylation in monozygotic twins discordant for schizophrenia. Neurochem. Int. 56, 906–910. 10.1016/j.neuint.2010.03.018 20371257

[B49] Stefansson H. Sigurdsson E. Steinthorsdottir V. Bjornsdottir S. Sigmundsson T. Ghosh S.. (2002). Neuregulin 1 and susceptibility to schizophrenia. Am. J. Hum. Genet. 71, 877–892. 10.1086/342734 12145742 PMC378543

[B50] Tan H. Y. Nicodemus K. K. Chen Q. Li Z. Brooke J. K. Honea R.. (2008). Genetic variation in AKT1 is linked to dopamine-associated prefrontal cortical structure and function in humans. J. Clin. Invest. 118, 2200–2208. 10.1172/JCI34725 18497887 PMC2391279

[B51] Walshe M. Vassos E. Picchioni M. Shaikh M. Toulopoulou T. Collier D.. (2012). The association between COMT, BDNF and NRG1 and Premorbid social functioning in patients with psychosis, their relatives and controls. Scientifica (Cairo) 2012:560514. 10.6064/2012/560514 24278715 PMC3820633

[B52] Zhao Z. Ksiezak-Reding H. Riggio S. Haroutunian V. Pasinetti G. M. (2006). Insulin receptor deficits in schizophrenia and in cellular and animal models of insulin receptor dysfunction. Schizophr. Res. 84, 1–14. 10.1016/j.schres.2006.02.009 16581231

